# Osteosarcoma-Specific Genes as a Diagnostic Tool and Clinical Predictor of Tumor Progression

**DOI:** 10.3390/biology11050698

**Published:** 2022-05-01

**Authors:** Pattaralawan Sittiju, Parunya Chaiyawat, Dumnoensun Pruksakorn, Jeerawan Klangjorhor, Weerinrada Wongrin, Phichayut Phinyo, Rawikant Kamolphiwong, Areerak Phanphaisarn, Pimpisa Teeyakasem, Prachya Kongtawelert, Peraphan Pothacharoen

**Affiliations:** 1 Thailand Excellence Center for Tissue Engineering and Stem Cells, Department of Biochemistry, Faculty of Medicine, Chiang Mai University, Chiang Mai 50200, Thailand; pattaralawan.s@gmail.com (P.S.); prachya.kongtawelert@gmail.com (P.K.); 2 Musculoskeletal Science and Translational Research Center, Department of Orthopedics, Faculty of Medicine, Chiang Mai University, Chiang Mai 50200, Thailand; parunya.chaiyawat@cmu.ac.th (P.C.); dumnoensun.p@cmu.ac.th (D.P.); jeerawan.klangjorhor@gmail.com (J.K.); phichayutphinyo@gmail.com (P.P.); aphanphaisarn@hotmail.com (A.P.); pimpis.mk@gmail.com (P.T.); 3 Center of Multidisciplinary Technology for Advanced Medicine (CMUTEAM), Faculty of Medicine, Chiang Mai University, Chiang Mai 50200, Thailand; 4 Department of Statistics, Faculty of Science, Chiang Mai University, Chiang Mai 50200, Thailand; weerinradaj@gmail.com; 5 Center for Clinical Epidemiology and Clinical Statistics, Faculty of Medicine, Chiang Mai University, Chiang Mai 50200, Thailand; 6 Department of Family Medicine, Faculty of Medicine, Chiang Mai University, Chiang Mai 50200, Thailand; 7 Department of Biomedical Sciences and Biomedical Engineering, Faculty of Medicine, Prince of Songkla University, Songkhla 90110, Thailand; k.rawikant@gmail.com

**Keywords:** osteosarcoma, biomarker, mRNA expression, qRT-PCR

## Abstract

**Simple Summary:**

The standard method for the diagnosis and monitoring of osteosarcoma is biopsy and tumor imaging, which causes discomfort to patients and is difficult to repeat. A blood sample can be used as a non-invasive method for monitoring tumor material. Vimentin and ezrin show clinical significance in samples obtained from OS patients but need circulating tumor cell purification, since they are expressed in leukocytes. Due to the low-temperature storage of the samples, it proved impossible to perform purification to remove the contamination. We propose that novel or OS-specific biomarkers using differential gene expression from the Gene Expression Omnibus (GEO) database is a promising approach for developing diagnostic and tumor progression strategies. Seven genes from the database showed significant expression in OS cell lines/primary cells compared to a normal blood donor, together with *ezrin* and *VIM*. The expression of the five candidate genes together with ezrin and vimentin were quantified by qRT-PCR and analyzed using a mathematical model with high efficiency to discriminate between OS patients and normal samples, resulting in the selection of three candidate genes: *COL5A2* (one of the five from the database) as well as *ezrin* and *VIM*. Our study demonstrates that these genes in retrospective samples could serve as tools of OS detection and predictors of disease progression.

**Abstract:**

A liquid biopsy is currently an interesting tool for measuring tumor material with the advantage of being non-invasive. The overexpression of vimentin and ezrin genes was associated with epithelial-mesenchymal transition (EMT), a key process in metastasis and progression in osteosarcoma (OS). In this study, we identified other OS-specific genes by calculating differential gene expression using the Gene Expression Omnibus (GEO) database, confirmed by using quantitative reverse transcription-PCR (qRT-PCR) to detect OS-specific genes, including *VIM* and *ezrin* in the buffy coat, which were obtained from the whole blood of OS patients and healthy donors. Furthermore, the diagnostic model for OS detection was generated by utilizing binary logistic regression with a multivariable fractional polynomial (MFP) algorithm. The model incorporating *VIM*, *ezrin*, and *COL5A2* genes exhibited outstanding discriminative ability, as determined by the receiver operating characteristic curve (AUC = 0.9805, 95% CI 0.9603, 1.000). At the probability cut-off value of 0.3366, the sensitivity and the specificity of the model for detecting OS were 98.63% (95% CI 90.5, 99.7) and 94.94% (95% CI 87.5, 98.6), respectively. Bioinformatic analysis and qRT-PCR, in our study, identified three candidate genes that are potential diagnostic and prognostic genes for OS.

## 1. Introduction

Osteosarcoma (OS), although relatively rare, is the most common primary malignancy of the bone, with a worldwide incidence of 3.4 cases per million people per year [1]. OS is found predominantly in the second decade of life [1,2]. Forty percent of OS patients with tumors metastasized to secondary sites were found to have a poor treatment response and poor recovery, even when combination therapies were employed [3].

For metastatic OS, magnetic resonance imaging (MRI), computed tomography (CT), and positron emission tomography (PET) are the standard methods for diagnosis and follow-up monitoring. A PET-CT is more sensitive for bone metastasis detection than scintigraphy, which is currently the standard method for metastatic bone lesion detection [4,5]. Even so, pulmonary nodules smaller than 5–9 mm are still undetectable by PET/PET-CT [1,6,7]. The sensitivity of the evaluation of bone metastasis is increased when scintigraphy is combined with PET-CT [5].

Liquid biopsy is an alternative technique for predicting metastasis, representing a promising approach for diagnostic, prognostic, and personalized therapeutic purposes. Among liquid biopsy biomarkers, circulating tumor cells (CTCs) represent a propitious avenue for identifying cancer metastasis. Owing to the novelty and complexity of the methods, these techniques are not widely used [8]. Cancer-specific mRNA analysis is one encouraging approach for tracing cancer cells in the blood; however, specific mRNA markers for OS are not generally established [9,10]. Comparative expression analysis using information from biodata resources is a new pregenital approach for the identification of tumor-specific markers. Among the sources of bioinformatics data, the Gene Expression Omnibus (GEO) has been widely adopted for identifying tumor-specific genes [11,12].

Epithelial-mesenchymal transition (EMT) plays a key role in tumor metastasis [13]. Vimentin, an intermediate filament, which is considered to be a marker of EMT, is high in both mRNA and protein levels during intermediate metastasis processes, and its overexpression is associated with a poor prognosis in several sarcomas, including OS [14,15,16]. Nevertheless, vimentin is intracellularly expressed in normal mesenchymal cells, such as white blood cells (WBC) and connective tissue cells, limiting its applicability as a tumor marker [17,18]. Ezrin, a membrane cytoskeletal-linker protein of the ezrin/radixin/moesin (ERM) family, is a key regulator in the progression and metastasis of OS [19]. Correlation for the overexpression of ezrin and CTCs with an EMT phenotype was also reported in OS [10].

To predict the progression of OS, we propose a simple and inexpensive method that identifies the novel OS-specific genes in blood samples. Candidate OS-specific genes were identified using comparative gene expression analyses datasets from the gene expression database, and their expression was then analyzed in the buffy coat samples of 73 OS patients and 79 healthy donors, using the widely available quantitative reverse transcription-PCR (qRT-PCR) method.

## 2. Materials and Methods

### 2.1. Patients

**Figure 1:**
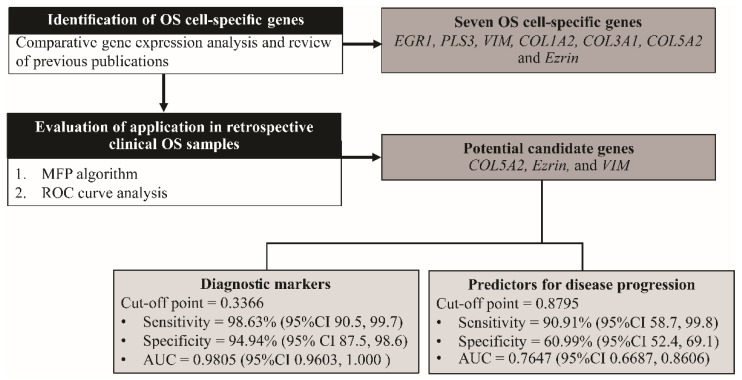
Overview of the study and workflow of methods. OS, osteosarcoma; *COL1A2*, collagen type-I alpha-2 chain; *PLS3*, plastin-3; *COL5A2*, collagen type-V alpha-2 chain; *COL3A1*, collagen type-III alpha-1 chain; *EGR1*, early growth response protein 1; *VIM*, vimentin; MFP; multivariable fractional polynomial, ROC; receiver operating characteristic curve; AUC, area under the ROC curve; CI, confidence interval.

Ethylenediaminetetraacetic acid (EDTA) whole-blood samples and tumor tissue samples were retrospectively collected from 62 stage-IIB and 11 stage-III patients during diagnostic procedures conducted between 2012 and 2020 at Maharaj Nakorn Chiang Mai Hospital. The EDTA-buffy coat (500 μL) was gathered from residual anonymous samples of 91 healthy individuals during donor-screening procedures, obtained from residuals that would otherwise have been discarded, during the blood-component preparation process at the Blood Bank Section of Maharaj Nakorn Chiang Mai Hospital. Informed consent was obtained from the patients or parents, and/or legal guardians, in case of participants under the age of 18 years (including donors of tissue samples). All the blood and tissue samples were collected after receiving approval from the Research Ethics Committee, Faculty of Medicine, Chiang Mai University (ORT-2557-02717 and ORT-2562-06549). All the methods were performed according to the relevant guidelines and regulations. An overview of the study and the workflow of methods is shown in Figure 1.

### 2.2. Bioinformatic Analysis

An Affymetrix HG-U133Plus2.0 DNA microarray (Platform GPL570) of OS cell lines (GSE70414, GSE30807, GSE37552, GSE18947, GSE16089, GSE7454, GSE41828, GSE46493, GSE41445, and GSE55957), primary OS cells (GSE85537), and the whole blood of healthy people (GSE93272) who represented the buffy coat composition were retrieved from the GEO. All data were from cells not treated with any agent or vector. Accession codes are given in Table S1. The gene expression analysis to identify candidate genes was performed by comparing (1) OS cell lines with healthy whole-blood samples, and (2) primary OS cells with healthy whole-blood samples. The robust multi-array average (RMA) algorithm, through a custom brain array chip description file (CDF, ENTREZG, V19), was used to calculate the quantile normalization background adjustment and summarized as previously described [11]. For the investigation of differential gene expression, *p*-values were calculated with Linear Models for Microarray (limma) data in R. A *p*-value with a log2 expression ratio (ER) greater than two was set as the cut off for the initial selection of candidate genes [20].

### 2.3. Sample Preparation

Healthy peripheral-blood mononuclear cells (PBMCs): Buffy coat samples from the blood-component separation process were diluted with phosphate-buffered saline (1:1). Gradient centrifugation using Lymphoprep™ (STEMCELL Technologies, Vancouver, BC, Canada) was employed for PBMCs’ isolation. The PBMCs were collected and counted under a light microscope with a hemocytometer.

Buffy coat: EDTA whole-blood specimens were centrifuged at 1600× *g* for 15 min; the buffy coat layer between packed red blood cells and plasma was collected and stored at −80 °C. The cryopreserved buffy coat samples from OS patients and healthy donors were lysed with lysis buffer RA1 (Macherey Nagel, Düren, Germany).

Primary cells: Primary cells were grown in Dulbecco’s modified Eagle’s medium (DMEM) with 10% (*v*/*v*) fetal bovine serum and maintained in a humidified atmosphere of 37 °C with 5% CO_2_ [21].

### 2.4. qRT-PCR

Total RNA was extracted with an Illustra RNAspin Mini Kit (GE Healthcare Europe GmbH, Freiburg, Germany), and cDNAs were generated by iScript^TM^ (Bio-Rad, Hercules, CA, USA). The PCR reactions were performed with an Applied Biosystems 7500/7500 Fast Real-Time PCR system using SensiFAST™ SYBR^®^ Lo-ROX (Bio-Rad, Hercules, CA, USA) for 45 cycles. Each cycle was performed as follows: 5 s at 95 °C, 10 s at 60 °C, and then 30 s at 72 °C. The RNA levels of candidate genes (*COL5A2, ezrin*, and *VIM*) in primary OS cells and normal PBMCs were normalized with beta-actin (ACTB) as a housekeeping gene and calculated using the 2^(−ΔC (T))^ method. RNA levels of candidate genes (*COL1A2, PLS3, COL5A2, COL3A1, EGR1, ezrin*, and *VIM*) in all buffy coat samples, pinpointed from both bioinformatic analysis and previous publications, were normalized based on global mean strategy using qBasePlus version 3.3 software. Raw Ct data were imported into software analyzer. Ct values over 35 were excluded before analysis. The relative expression of candidate genes were qualified with calculated geometric mean as a normalization factor [22]. The primer sequences are listed in Table 1.

### 2.5. Statistical Analysis

Statistical analysis of the relative expression of candidate genes was performed using SPSS 22.0 (IBM Corp., Armonk, NY, USA), Stata 16 (StataCorp, College Station, TX, USA), and Prism 8.4.3 (GraphPad, La Jolla, CA, USA). The data are shown as the mean  ±  standard deviation (SD). The significance of the difference between the means of OS patients and healthy donors was determined using the Mann–Whitney U-test for the ordinal or continuous data that were not normally distributed. *p*-values less than 0.05 were considered statistically significant.

Candidate genes were selected through a retrospective study. Non-parametric regression and multivariable modeling were constructed with data from both OS patients and healthy donors using fractional polynomials. We explored the shape of the association between relative gene expression and log odds of osteosarcoma using locally weighted scatter-plot smoothing (LOWESS) and fractional polynomial plots. A diagnostic model for the prediction of OS and OS metastasis was derived using binary logistic regression with a multivariable fractional polynomial (MFP) algorithm to fit continuous determinants based on the actual shape of their association with the predicted endpoints [23,24]. The *p*-value cut off was set at 0.05 to exclude gene expression with a non-significant contribution from the equation model. The model discriminative ability was measured as the area under the receiver operating characteristic curve (ROC). Predicted probabilities of OS and OS metastasis were calculated using the model. Cut-off points for the diagnosis of OS and OS metastasis were established based on sensitivity and specificity.

## 3. Results

### 3.1. Identification of Osteosarcoma Cell-Specific Candidate Genes using Bioinformatics

To overcome the limitation of *VIM* and *ezrin*, i.e., their extensive expression in leukocytes, we identified OS-specific genes using the publicly available microarray gene expression datasets from the GEOs of OS cell lines (*n* = 29), primary OS cells (*n* = 3), and healthy whole-blood samples (*n* = 36), which were assumed to represent OS in circulation and blood cells in buffy coat samples. Datasets of samples that had been administered via any agent or vector were excluded. Quantile normalization background adjustment and summarization were calculated using a robust multi-array average (RMA) algorithm and a custom brain array chip description file (CDF, ENTREZG, V19). As described earlier, probe sets of genes and adjusted *p*-values were calculated using a limma package available in R to compare the gene expression.

**Figure 2:**
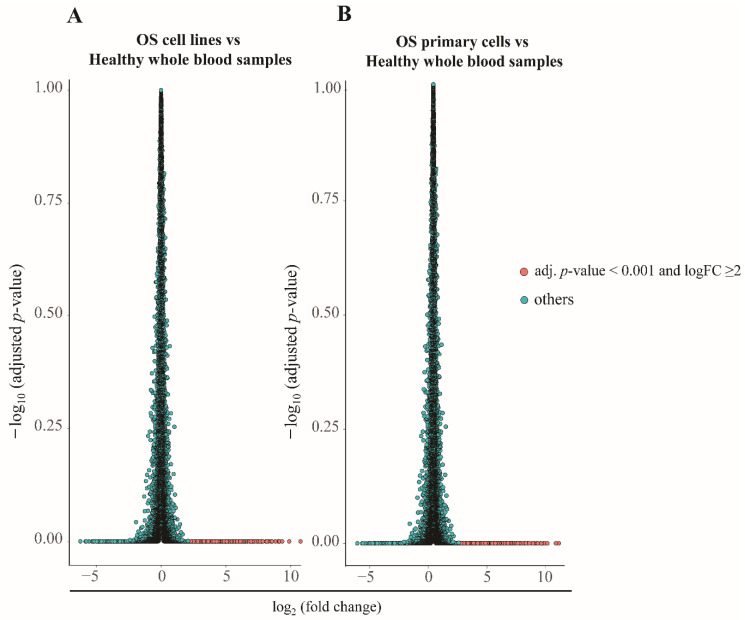
Comparative expression analysis. Volcano plots showing a pairwise comparison of gene expression in osteosarcoma (OS) cells (**A**) and OS primary cells (**B**) vs. healthy donors. The genes presented in red had an expression ratio of >2 (log_2_) and adjusted *p*-value < 0.01.

**Figure 3:**
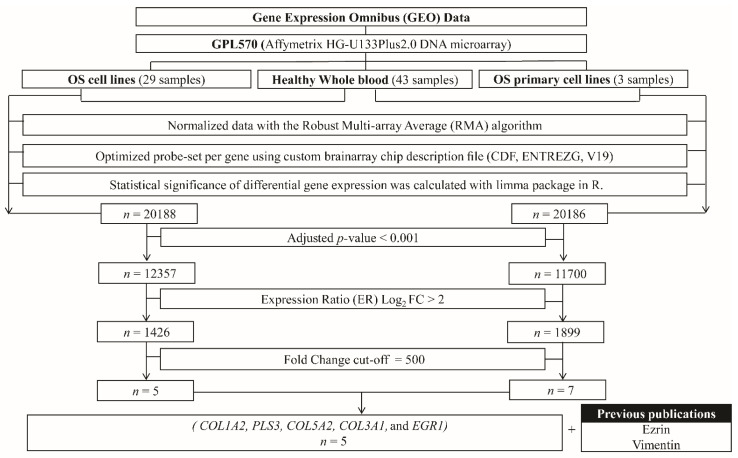
Diagram of the selection procedure for candidate genes. OS, osteosarcoma; *COL1A2*, collagen type-I alpha-2 chain; *PLS3*, plastin-3; *COL5A2*, collagen type-V alpha-2 chain; *COL3A1*, collagen type-III alpha-1 chain; *EGR1*, early growth response protein 1; *VIM*, vimentin.

In the GEO data, 20,188 and 20,186 genes had been reported in OS cell lines and primary OS cells, respectively. After calculation, we found significant upregulation of 1426 and 1899 genes in OS cell lines and primary OS cells, respectively (*p* < 0.001), when compared to healthy whole-blood cells, with a log_2_ expression ratio (ER) >2 (Figure 2A,B, respectively). These sets of genes were regarded as the upregulating genes. Among the upregulating genes, only five genes—*COL1A2, PLS3, COL5A2, COL3A1,* and *EGR1*—which presented a 500-fold change in expression in OS cell lines or primary OS cells compared to healthy whole-blood samples, were considered as the novel OS-specific genes (Figure 3).

### 3.2. Evaluation of the OS Diagnostic and Metastasis Predictive Potential of the OS-Specific Candidate Genes

**Figure 4:**
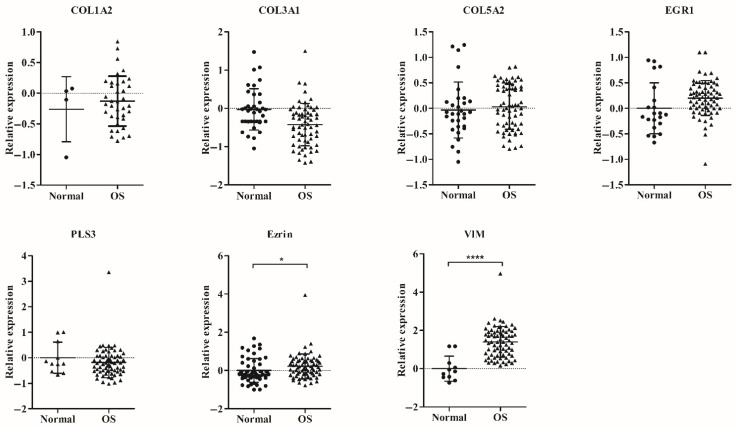
Detection of circulating osteosarcoma (OS) cells in the buffy coat of OS patients and healthy donors using qRT-PCR technique. Data are represented as scatter plot of *COL1A2, COL3A1, COL5A2, EGR1, PLS3, ezrin*, and *VIM* relative expression by qRT-PCR. The expressions of seven candidate genes in OS patients (*n* = 73) and healthy donors (*n* = 79) were quantified using qRT-PCR. Each data expression was normalized to the global mean analyzed in triplicate. Only data with Ct values below 35 are displayed as vertical scatter plot; bars represent the mean ± SD. The Mann–Whitney U-Test test was used to determine the *p*-values, * *p* < 0.05, **** *p* < 0.0001.

According to the involvement of *VIM* and *ezrin* in EMT and metastasis, we evaluated their expression together with newly identified OS-specific genes. The samples from OS patients (*n* = 73) and healthy donors (*n* = 79) (clinical characteristics shown in Table 2) were evaluated for expression of the candidate genes using qRT-PCR; the Ct values of each of the candidate genes were normalized with that of the global mean. The analysis demonstrated that the relative expression levels of *ezrin* and *VIM* (*p* < 0.05) were significantly higher in OS patients than in healthy donors (Figure 4).

**Figure 5:**
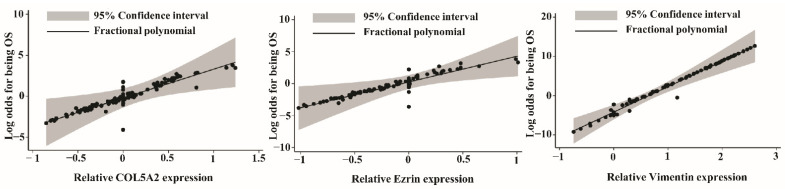
Locally weighted scatter-plot smoothing (LOWESS) and fractional polynomial assessment of the linear association between relative gene expression and log odds of osteosarcoma (fracplot of *COL5A2* was truncated minimal value, *Ezrin* and *VIM* were truncated maximal value for visualization).

The association between the relative expression of each candidate gene and the log odds of OS was non-linear (Figure 5). In the diagnostic model generated using the MFP algorithm, *COL5A2, ezrin*, and *VIM*, which showed significant contributions to the model, were included, while *COL1A2, COL3A1*, *EGR1,* and *PLS3* were excluded, as their *p*-values were less than 0.05 (data not shown). According to the diagnostic model, the probability of OS was calculated as follows:

$$probability\;of\;osteosarcoma=\frac{exp^{lp}}{1+exp^{lp}}\;(2.2.)$$

where *lp* was the linear predictor yielded from the formula:

linear predictor (lp)=constant+3.56 (COL5A2 FP term)+2.25 (Ezrin FP term)+6.13 (VIM FP term) (2.2.)

Each candidate gene term is referenced in Table 3.

**Figure 6:**
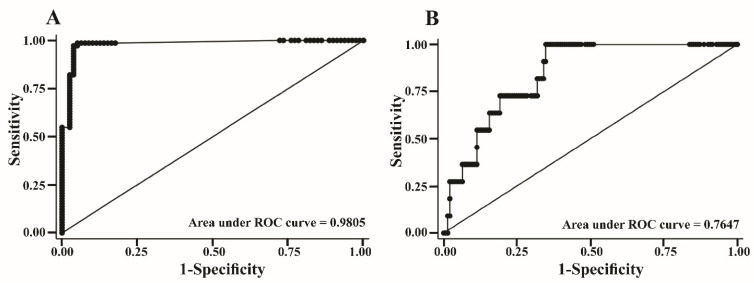
The ROC curve analysis was performed on the expression of *COL5A2*, *ezrin*, and *VIM* to examine the performance of the model for identifying OS in samples from OS patients and healthy donors (**A**) and the ability of these three genes to predict OS progression was evaluated (**B**).

ROC curve analysis was performed on the expression of *COL5A2,* e*zrin*, and *VIM* in samples from OS patients and healthy donors to examine the diagnostic performance of the model for identifying OS (Figure 6A). At the probability cut off of 0.3366, the sensitivity was 98.63% (95% CI 90.5, 99.7) and the specificity was 94.94% (95% CI 87.9, 99.6), with an area under the ROC curve of 0.9805 (95% CI: 0.87.5, 98.6) (Table 4). Based on the derived MFP model, we further evaluated the ability of *COL5A2*, *ezrin*, and *VIM* to predict metastatic OS. Almost-stage-III OS samples were positive at the cut-off point of 0.8795 with 90.91% sensitivity (95% CI 58.7, 99.8), 60.99% specificity (95% CI 52.4, 69.1), and a 0.7647 area under the ROC curve (95% CI 0.6687, 0.8606) (Figure 6B).

### 3.3. Expression of Candidate Genes in Primary OS Cells from OS Patients in Comparison with Healthy Donors’ PBMCs

**Figure 7:**
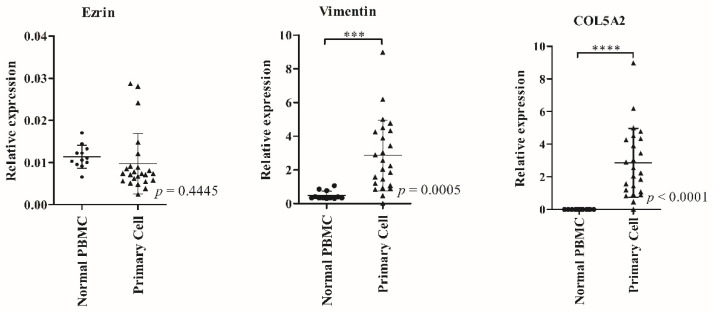
Comparison of the expression levels of three osteosarcoma (OS)-specific candidate genes between primary OS cells (*n* = 24) and normal PBMCs (*n* = 12) using the qRT-PCR technique. The relative expression levels of *COL5A2*, *ezrin*, and *VIM* were quantified by qRT-PCR. Expression data were normalized to the β-actin RNA level by the 2^−∆Ct^ method. Each sample was analyzed in triplicate. Data are displayed as vertical scatter plots with bars representing the mean ± SD. The Mann–Whitney U-Test test was used to determine *p*-values, *** *p* < 0.001, **** *p* < 0.0001.

We further explored whether the three candidate genes (*COL5A2, ezrin*, and *VIM*) were highly specific to clinical OS tumor origin. Further to this, we analyzed the mRNA expression of these candidate genes in primary OS cells from OS patients (*n* = 24) and PBMCs from healthy donors (*n* = 12) using the qRT-PCR method (clinical characteristics shown in Table S2). The results showed that the expressions of *COL5A2* and *VIM* in the primary OS cells were significantly higher than those in normal PBMCs, whereas *ezrin* expression was non-significantly different (*p* < 0.05) (Figure 7).

## 4. Discussion

Many previous studies demonstrated that the upregulation of e*zrin* and *VIM* is associated with the OS metastatic stage. Ezrin, a cross-linker protein, plays an essential role in many metastatic phenotypes of cancer, including pediatric sarcomas, OS, and rhabdomyosarcoma [25]. The expression level of ezrin was high in OS circulating cells, especially in OS metastatic stage-III in the Enneking staging system [10]. Vimentin, a mesenchymal marker, has recently been reported to be an indicator of the epithelial-to-mesenchymal transition (EMT), associated with migration and metastasis in various cancers as well [17,26]. The overexpression of vimentin is demonstrated in human OS tumor tissue [27]. However, several previous studies reported that *VIM* and *ezrin* expression were also found in normal cells, including leucocytes, which limits their usage as a marker for OS. The samples for gene expression analysis in our study were prepared as a buffy coat. The total RNA was extracted from the whole-buffy coats without further enrichment by isolating CTCs, since the RNAs from the frozen cells would otherwise be lost after thawing, following the injury of the frozen cells by ice crystallization, as the buffy-coat samples were frozen at −80 °C without preservatives [28]. To avoid losing the total RNAs of the circulating OS cells and reduce the number of enrichment steps, total RNA from whole-buffy coats was extracted immediately after thawing. Consequently, other interfering genetic components—the ones that were not derived from circulating OS cells, especially leukocyte RNAs—could not be discarded in this study.

In this study, we attempted to overcome the limitation of *VIM* and *ezrin* by selecting OS-specific genes from the differences in gene expression between OS cell lines or primary OS cells and healthy donor cells; here, we identified significant upregulation of five novel OS-specific genes (*COL1A2, PLS3, COL5A2, COL3A1*, and *EGR1*). Most of them, including *COL1A2, COL5A2, EGR1*, and *COL3A1*, have been previously reported as upregulated genes in OS tissues when compared to normal tissues, and the translated proteins of those genes are associated with OS progression [29,30,31,32,33]. In addition, high expression of *PLS3* was also related to tumor progression, in several types of tumors [34,35,36,37,38].

Before we investigated candidate gene expression in the specimens, we used reference genes to normalize the expression level of a candidate gene in the OS-spike sample in PBMC using *ACTB*, *GAPDH* (glyceraldehyde 3-phosphate dehydrogenase) and *B2M* (β2-Microglobulin). The results found that the expression levels were not consistent with the OS cell number (data not shown) and we, therefore, decided to apply the normalization strategy of global mean to determine relative expressions of candidate genes in specimens in this study. According to previous studies, normalization with global mean found it not only reduced technical error but also increased the accuracy of the expression level of a large number of genes [39,40]. In this study, we evaluated normalization performance between not normalized and global normalized data by calculating cumulative distributions (CV) of the standard deviation (SD) for each individual gene, across all buffy coats within a group. Our data showed that the normalized data reduced the overall variation within a group (Figure S2).

Measurement of candidate gene expression, in clinical samples from OS patients and healthy donors, showed statistically significant differences in the expression of *ezrin* and *VIM* genes between the two groups (*p* < 0.05), but not in the expression of *COL1A2, COL3A1, COL5A2,* and *EGR1* genes (Figure 4). Not surprisingly, OS exhibited high heterogeneity and complexity for the genomic and expression levels between patients [41]. The efficiency of each candidate gene in the diagnosis of OS was evaluated with binary logistic regression, based on the MFP algorithm using relative expression data. The model that included *VIM, ezrin,* and *COL5A2* performed the best at discriminating OS samples from healthy donor samples. The same model also exhibited the ability to predict OS metastasis at a probability cut-off value of 0.8795. Due to the small sample size of stage-III patients, it is unclear whether these three genes are biomarkers against metastasis. However, our findings exhibit a static relationship between the expression of candidate genes and disease progression. To study this issue further, collecting data for a larger sample size will be necessary in future experiments.

Due to the limitation of the samples, it was not possible to isolate circulating OS cells from the frozen buffy coat. To prove whether the measured candidate genes originated from OS cells in the bloodstream, the expression levels of *COL5A2, ezrin*, and *VIM* were analyzed in primary OS cells with qRT-PCR, and PBMCs from healthy donors were used as the control cells. Among the three candidate genes, the expression levels of *COL5A2, PLS3*, and *VIM*—all except *ezrin*—in OS cells were significantly higher than those of PBMC (*p* < 0.05) (Figure 7). *COL5A2* is classified as a clinical biomarker for metastasis, such as gastric, colorectal, and bladder cancer, including osteosarcoma, and has been associated with the development of tumors in the immune system, proliferation, and angiogenesis [22,33,42,43,44]. In our study, a significantly higher expression level of *COL5A2* was found in OS patients when compared to healthy donors. Thus, *COL5A2* could be a novel liquid biopsy marker for prognosis prediction in OS. On the other hand, *ezrin*, which has been reported as a typical EMT marker, showed a significantly high expression in the frozen OS buffy coats, but not in primary OS cells. Since there was no difference in *ezrin* expression between OS primary cells and PBMCs, we supposed that the population of clusters of tumor cells with the potential to become CTCs was low in the primary OS tumors examined in this study. However, the expression status of *ezrin*, the EMT marker, in primary cells might not necessarily be an indication of CTC clusters inside primary tumors, especially before EMT changes begin. A previous study indicated that high mRNA and protein levels of *ezrin* in clinical ovarian carcinoma (OC) specimens of malignant effusions were observed when compared to solid tumors, including primary tumors and solid metastases. Moreover, overexpression of ezrin was found in spheroid OC cells compared to their three-dimensional alginate scaffold [45]. A study by Kim et al. showed that almost 50 percent of OS tumor specimens did not express ezrin [46], while more than 70 percent of CTCs were positive among 38 OS patients through RNA-ISH [10]. However, a high expression of ezrin, both in RNA and protein levels, in OS patients, was positively correlated with the metastatic stage and OS recurrence [47,48]. Thus, our study showed that the expression of *ezrin* was significantly higher in all PBMC specimens compared to an OS cell line under suspended conditions, but not in adherent conditions, as shown in the Supplementary Material (Figure S1). Unfortunately, this experiment’s sample size was small, meaning we cannot conclude there is a definite relationship between the histologic subtype and ezrin expression.

Finally, comparative expression analysis of genes between single circulating tumor cells and other circulating blood cells could be further investigated. As none of the fresh blood samples were from OS patients, we could not directly conclude whether these three genes were exactly from OS circulating cells. Looking ahead, these three predictors could be further evaluated to monitor disease progression, and for the prediction of therapeutic response and tumor recurrence, in a larger sample size. This could potentially improve the predictive tools indicating OS progression.

## 5. Conclusions

This study demonstrated the feasibility of using *VIM, ezrin, and COL5A2*, detectable through qRT-PCR, as potential candidate biomarkers to detect OS-specific mRNA in an OS sample. This gene set identifies the OS cells in circulation and, thus, could be used as a diagnostic and disease-progression tool for OS.

## Figures and Tables

**Table 1:** Real-time PCR primer sequences.

Gene Group	Name	Sequence (5′-3′): Forward (F); Reverse (R)	Accession Number
Comparative expression analysis	*COL1A2*	F: AGGGCAACAGCAGGTTCACTTA R: TCAGCACCACCGATGTCCAA	NM_000089.4
	*PLS3*	F: TTGTCCAGCGAAGGAACACA R: ACAGGTCATCGGTGTTAGGG	NM_001172335.3
	*COL5A2*	F: CGCTTTTGTCATGTCAGTGGTT R: GTGTCATGTTGCCTTTGTGGG	NM_000393.5
	*COL3A1*	F: CGCCCTCCTAATGGTCAAGG R: TTCTGAGGACCAGTAGGGCA	NM_000090.4
	*EGR1*	F: TCCCATTTACTCAGCGGCAC R: TGGAAACAGGTAGTCGGGGA	NM_001964.3
Previous study source	*Ezrin*	F: AAGGGTTCTGCTCTGACTCCA R: TGGTTTCGGCATTTTCGGTT	NM_003379.5
	*VIM*	F: TCTCTGAGGCTGCCAACCG R: CGAAGGTGACGAGCCATTTCC	NM_003380.5
Housekeeping gene	*ACTB*	F: GTAAAGACCTCTATGCCAACA R: GGACTCATCGTACTCCTGCT	NM_031144.3

**Table 2:** Clinical characteristics of OS patients and healthy donors (buffy coat samples).

Parameters		OS Patients (*n* = 73)	Healthy Donors (*n* = 79)
Median age (range)	Childhoods and adolescents	14 (5–24), (71.2%)	22 (18–24), (46.8%)
	Adults	57 (25–75), (28.8)	42 (25–55), (53.2%)
Gender	Male	36 (49.3%)	52 (65.8%)
	Female	37 (50.7%)	27 (34.2%)
Enneking stage	IIB	62 (84.9%)	-
	III	11 (15.1%)	-
Tumor location	Femur Tibia	35 (47.9%) 15 (20.5%)	- -
	Other	23 (31.5%)	-
Metastasis	Bone	3 (4.1%)	-
	Lung	6 (8.2%)	-
	Bone and Lung	2 (2.7%)	-
	None	62 (84.9%)	-

**Table 3:** Multivariable fractional polynomial logistic regression model for OS diagnosis.

Candidate Gene	Covariate Transformation		Β	95% CI	*p*
	df	FP Term after MFP Transformation			
*Intercept*	-	-	0.546	−0.452, 1.544	-
*COL5A2*	1	*COL5A2* + 0.489473678	4.523	2.036, 7.011	<0.0001
*Ezrin*	1	*Ezrin* + 0.080723685	2.801	0.363, 5.238	0.024
*VIM*	1	*VIM–*0.6512500006	6.247	4.251, 8.242	<0.0001

**Table 4:** Diagnostic and metastatic prediction accuracy (*n* = 152).

Clinical Character (*n*)			Probability Cut-Off Point	AUC (95% CI)	Sensitivity (95%CI)	Specificity (95%CI)	LHR + (95%CI)
UD	Normal	OS	0.3366	0.9805	98.63%	94.94%	19.21
26	57	69		(0.9603, 1.0000)	(90.5, 99.7)	(87.5, 98.6)	(7.39, 49.95)
UD	Non-metastasis (Normal and IIB)	Metastasis (III)	0.8795	0.7257	100%	54.78%	2.33
26	115	11		(0.621, 0.8302)	(71.5, 100.0)	(45.2, 64.1)	(1.76, 3.08)

## Data Availability

Data is contained within the article.

## Supplementary Materials

The following supporting information can be downloaded at: https://www.mdpi.com/article/10.3390/biology11050698/s1, Figure S1. Comparison of the expression levels of ezrin between normal PBMCs, SaOS-2 (human OS cell lines), in the attached conditions and anchorage-independent conditions. The relative expression of ezrin was quantified by qRT-PCR in 12 samples of normal PBMCs and SaOS-2. Expression data were normalized on the β-actin RNA level by the 2^−∆Ct^ method. Each sample was analyzed in triplicate. Data are displayed as vertical scatter plots with bars representing the mean ± SD. The one-way ANOVA was used to determine *p*-values: *** *p* < 0.0001; Figure S2. Cumulative distributions (CV) of the standard deviation (SD). Standard deviations (SDs) for each individual gene in the heathy and OS buffy coat samples presented not normalized (- -) and global mean normalized expression data (—); Table S1: The microarray expression datasets used in this study; Table S2. Clinical characteristics of OS patients and healthy donors (primary cells and PBMCs, respectively).
