# Supplementary material for: Osteosarcoma-Specific Genes as a Diagnostic Tool and Clinical Predictor of Tumor Progression
Source: Biology (Basel). 2022 May 1;11(5):698. doi: 10.3390/biology11050698 (PMC9138411; doi:10.3390/biology11050698)
Supplement: Supplementary file 1 [file biology-11-00698-s001.zip › biology-1616875-supplementary.pdf]

Table S1. The microarray expression data sets used in this study are publicly available (continued).

Entity	GEOAccession codes		Title of Samples
	Series	Sample	
OS cell line (n=29)	GSE70414	GSM1727193<br>GSM1727194<br>GSM1727195<br>GSM1727196<br>GSM1727197	1. MG63 [mRNA]<br>2. Saos [mRNA]<br>3. HOS [mRNA]<br>4. NY [mRNA]<br>5. Hu09 [mRNA]
	GSE30807	GSM764200	6. osteosarcoma U2OS cells
	GSE37552	GSM921514<br>GSM921515<br>GSM921516<br>GSM921517	7. Human_Osteosarcoma_Non-Metastatic_SaOS-2_Parental Line<br>8. Human_Osteosarcoma_ Metastatic_LM7_Subline of SaOS-2<br>9. Human_Osteosarcoma_Non-Metastatic_HOS_Parental Line<br>10. Human_Osteosarcoma_ Metastatic_143B_Subline of HOS
	GSE18947	GSM469260<br>GSM469261<br>GSM469264	11. low metastatic potential cell subline of Sosp-9607, biological rep1<br>12. low metastatic potential cell subline of Sosp-9607, biological rep2<br>13. low metastatic potential cell subline of Saos-2
	GSE16089	GSM402655<br>GSM402656<br>GSM402657	14. Saos-2 parental cells replicate 1<br>15. Saos-2 parental cells replicate 2<br>16. Saos-2 parental cells replicate 3
	GSE7454	GSM180459	17. U-2OS (no-treatment) expression profile (AffyChip U133 Plus 2.0 platform)
	GSE41828	GSM1025090<br>GSM1025091<br>GSM1025092<br>GSM1025093<br>GSM1025094	18. U2OS, Untreated 24 hours rep1<br>19. U2OS, Untreated 24 hours rep2<br>20. U2OS, Untreated 24 hours rep3<br>21. U2OS, Untreated 24 hours rep4<br>22. U2OS, Untreated 24 hours rep5
	GSE46493	GSM1131226<br>GSM1131227<br>GSM1131228	23. U2OS No Treatment, Replicate 1<br>24. U2OS No Treatment, Replicate 2<br>25. U2OS No Treatment, Replicate 3

Table S1. *Cont.*

Entity	GEOAccession codes		Title of Samples
	Series	Sample	
OS cell line (n=29)	GSE41445	GSM1017514<br>GSM1017515<br>GSM1017516	26. U2OS_21a<br>27. U2OS_21b<br>28. U2OS_21c
	GSE55957	GSM1349294	29. ZOS osteosaracoma cell line
Primay OS cells (n=3)	GSE85537	GSM2276632<br>GSM2276633<br>GSM2276634	30. Bone1, biological rep1<br>31. Bone2, biological rep2<br>32. Bone3, biological rep3
Healthy whole blood (n=43)	GSE93272	GSM2449608<br>GSM2449609<br>GSM2449610<br>GSM2449611<br>GSM2449612<br>GSM2449613<br>GSM2449614<br>GSM2449615<br>GSM2449616<br>GSM2449617<br>GSM2449618<br>GSM2449619<br>GSM2449620<br>GSM2449621<br>GSM2449622<br>GSM2449623<br>GSM2449624<br>GSM2449625<br>GSM2449626<br>GSM2449627<br>GSM2449628	33. Whole blood from healthy control(HC003_1)<br>34. Whole blood from healthy control(HC004_1)<br>35. Whole blood from healthy control(HC005_1)<br>36. Whole blood from healthy control(HC006_1)<br>37. Whole blood from healthy control(HC007_1)<br>38. Whole blood from healthy control(HC008_1)<br>39. Whole blood from healthy control(HC009_1)<br>40. Whole blood from healthy control(HC010_1)<br>41. Whole blood from healthy control(HC011_1)<br>42. Whole blood from healthy control(HC012_1)<br>43. Whole blood from healthy control(HC013_1)<br>44. Whole blood from healthy control(HC015_1)<br>45. Whole blood from healthy control(HC016_1)<br>46. Whole blood from healthy control(HC017_1)<br>47. Whole blood from healthy control(HC018_1)<br>48. Whole blood from healthy control(HC019_1)<br>49. Whole blood from healthy control(HC020_1)<br>50. Whole blood from healthy control(HC021_1)<br>51. Whole blood from healthy control(HC022_1)<br>52. Whole blood from healthy control(HC023_1)<br>53. Whole blood from healthy control(HC024_1

Table S1. *Cont.*

Entity	GEOAccession codes		Title of Samples
	Series	Sample	
Healthy whole blood (n=43)	GSE93272	GM2449629	54. Whole blood from healthy control(HC025_1)
		GSM2449630	55. Whole blood from healthy control(HC026_1)
		GSM2449631	56. Whole blood from healthy control(HC027_1)
		GSM2449632	57. Whole blood from healthy control(HC028_1)
		GSM2449633	58. Whole blood from healthy control(HC030_1)
		GSM2449634	59. Whole blood from healthy control(HC031_1)
		GSM2449635	60. Whole blood from healthy control(HC034_1)
		GSM2449636	61. Whole blood from healthy control(HC035_1)
		GSM2449637	62. Whole blood from healthy control(HC036_1)
		GSM2449668	63. Whole blood from healthy control(AU_2)
		GSM2449669	64. Whole blood from healthy control(AW_2)
		GSM2449670	65. Whole blood from healthy control(AY_2)
		GSM2449671	66. Whole blood from healthy control(BA_2)
		GSM2449672	67. Whole blood from healthy control(BB_2)
		GSM2449673	68. Whole blood from healthy control(HC003_2)
		GSM2449674	69. Whole blood from healthy control(HC005_2)
		GSM2449675	70. Whole blood from healthy control(HC008_2)
		GSM2449676	71. Whole blood from healthy control(HC023_2)
		GSM2449677	72. Whole blood from healthy control(HC025_2)
		GSM2449678	73. Whole blood from healthy control(HC026_2)
		GSM2449679	74. Whole blood from healthy control(HC034_2)
		GSM2449680	75. Whole blood from healthy control(HC036_2)

Table S2. Clinical characteristics of OS patients and healthy donors (primary cells and PBMCs, respectively).

**Parameters**		**OS patients (n=24)**	**Healthy donors (n=12)**
**Median age [range]**		19 [5–61]	22 [19–29]
**Gender**	Male	12 (50.0%)	5 (41.7%)
	Female	12 (50.0%)	7 (58.3%)
**Enneking stage**	IIB	19 (79.2%)	-
	III	5 (20.8%)	-
**Tumor location**	Femur	13 (51.2%)	-
	Tibia	6 (25.0%)	-
	Other	5 (20.8%)	-
**Metastasis**	Bone	1 (4.2%)	-
	Lung	3 (12.5%)	-
	Bone and lung	1 (4.2%)	-
	None	19 (79.2%)	

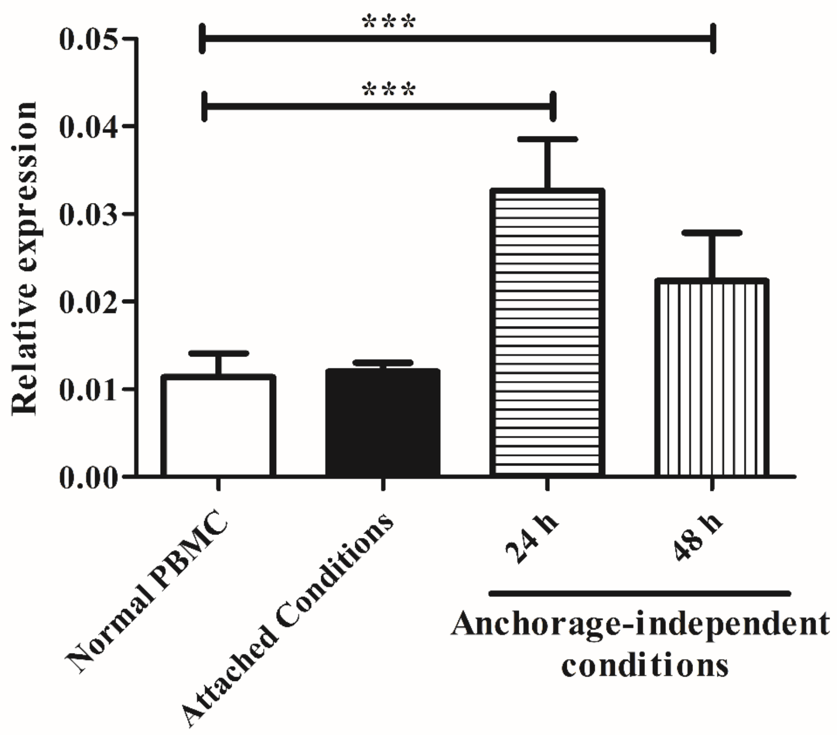

Figure S1. Comparison of the expression of ezrin among normal PBMCs, SaOS-2 (Human OS cell lines) in attached conditions and anchorage-independent conditions. The relative expression of *ezrin* was quantified by qRT-PCR in 12 samples of normal PBMCs and SaOS-2. Expression data were normalized on β-actin RNA level by the $2^{-\Delta Ct}$ method. Each sample was analyzed in triplicate. Data are displayed as vertical scatter plots with bars representing mean ± SD. The One-Way ANOVA test was used to determine *p*-values: ***, $p < 0.0001$.

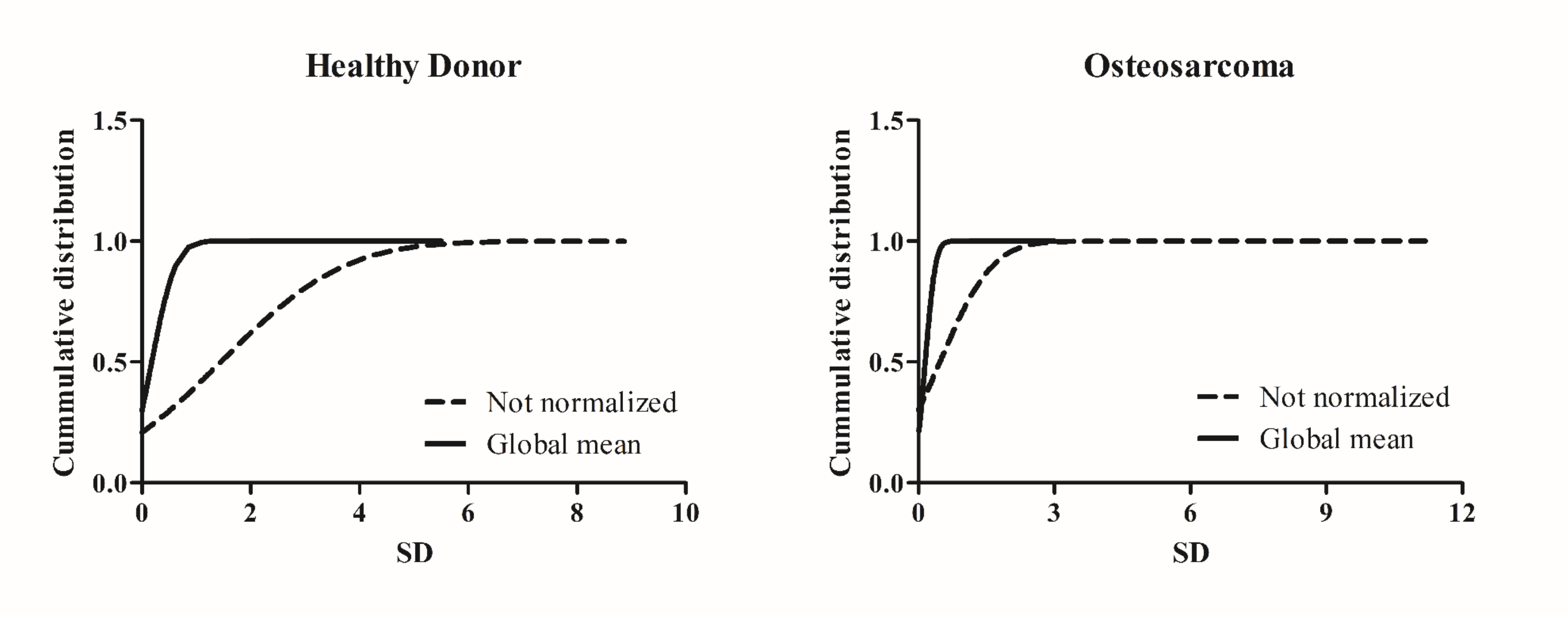

Figure S2. Cumulative distributions (CV) of the standard deviation (SD). Standard deviations (SDs) for each individual gene in the heathy and OS buffy coat samples presented not normalized (- -) and global mean normalized expression data (—).

Materials and Methods

Cell culture as anchorage-independent conditions

To mimic circulating tumor cell condition, Saos-2 osteosarcoma cell line was cultured on 6-well culture plate which coated with 450 µl of 6 mg/ml poly(2-hydroxyethyl methacrylate) or polyHEMA (Sigma-Aldrich, St. Louis, MO, USA) each well for 24 and 48 hours. The resistant osteosarcoma cells were observed under light microscope.
